# Longitudinal Monitoring of *EGFR* and *PIK3CA* Mutations by Saliva-Based EFIRM in Advanced NSCLC Patients With Local Ablative Therapy and Osimertinib Treatment: Two Case Reports

**DOI:** 10.3389/fonc.2020.01240

**Published:** 2020-07-24

**Authors:** Ning Li, Udayan Guha, Chul Kim, Leah Ye, Jordan Cheng, Feng Li, David Chia, Fang Wei, David T. W. Wong

**Affiliations:** ^1^Department of Oral and Maxillofacial Surgery, Xiangya Hospital, Central South University, Changsha, China; ^2^Thoracic and Gastrointestinal Oncology Branch, Center for Cancer Research, National Cancer Institute, Bethesda, MD, United States; ^3^School of Dentistry, University of California, Los Angeles, Los Angeles, CA, United States

**Keywords:** non-small cell lung cancer, osimertinib, local ablative therapy, gene mutation, acquired resistance, liquid biopsy

## Abstract

**Background:** The longitudinal monitoring of actionable oncogenes in circulating tumor DNA (ctDNA) of non-small cell lung cancer (NSCLC) is crucial for clinicians to evaluate current therapeutic response and adjust therapeutic strategies. Saliva-based electric field–induced release and measurement (EFIRM) is liquid biopsy platform to that can directly detect mutation genes with a small volume of samples. Herein, we compared the effectiveness of longitudinal monitoring for the combination of epidermal growth factor receptor (*EGFR)* and phosphatidylinositol-4,5-bisphosphate 3-kinase, catalytic subunit alpha (*PIK3CA)* mutations between saliva-based EFIRM and plasma-based platforms (ddPCR and NGS) in two advanced NSCLC patients undergoing the treatment with osimertinib before and after local ablative therapy (LAT).

**Patients and Methods:** Two patients with unresectable advanced NSCLC were enrolled into the National Institutes of Health Clinical Center (NIHCC) Study (ClinicalTrials.gov: 16-C-0092; local ablative therapy for the treatment of oligoprogressive, *EGFR*-mutated, non-small cell lung cancer after treatment with osimertinib). Serial collections of saliva, plasma, and metastatic tumor volume measurement by computed tomography (CT) were performed. Longitudinal paired saliva and plasma samples were analyzed for p.L858R *EGFR, exon19 del EGFR*, and p.E545K *PIK3CA* ctDNA using EFIRM (saliva) and ddPCR and NGS (plasma).

**Results:** In Case 1, the saliva ctDNA curve of *exon19 del EGFR* by EFIRM demonstrated a strong similarity to those of tumor volume (*R* = 0.78, *P* = 0.00) and *exon19 del EGFR* in ddPCR (*R* = 0.53, *P* = 0.01). Moreover, the curve of p.E545K *PIK3CA* in EFIRM showed similarity to those of tumor volume (*R* = 0.70, *P* = 0.00) and p.E545K *PIK3CA* in NGS (*R* = 0.72, *P* = 0.00). In Case 2, the curve of p.E545K *PIK3CA* in EFIRM revealed a reverse relationship to that of tumor volume (*R* = −0.65, *P* = 0.01).

**Conclusion:** In these two case reports, saliva-based EFIRM platform demonstrates a high level of concordance to plasma-based platforms (ddPCR and NGS) for longitudinally monitoring the combination of *EGFR* and *PIK3CA* ctDNA and can be a useful platform to monitor tumor progression and response to targeted therapy in NSCLC patients.

## Background

Somatic mutations in the epidermal growth factor receptor (*EGFR*) gene are detected in ~20% patients with non-small cell lung cancer (NSCLC) (1). *EGFR* tyrosine kinase inhibitors (TKIs) have significantly improved the response rate and survival in NSCLC patients harboring *EGFR*-sensitizing mutations (1). However, treatment with third-generation *EGFR* TKI (osimertinib) inevitably leads to acquired resistance (AR) because of the emergence of a osimertinib-resistant mutations (p.C797S) (2). Moreover, oncogene mutations such as *MET, BRAF*, and *PIK3CA*, have also been identified in NSCLC patients with AR to TKIs (3–5). Thus, due to the predictable development of the drug-resistant mutations, the detection, and longitudinal monitoring of TKI-related mutations in NSCLC are very important for clinicians to evaluate therapeutic response and adjust therapeutic strategies. There have been limited reports of longitudinal monitoring of NSCLC progression using *EGFR* and *PIK3CA* mutations.

Typically, tumor genomic information is acquired through thoracic tissue biopsies in procedures such as bronchial endoscopy or computed tomography (CT)-guided trans-mural punctures. However, since tissue biopsy are considered a surgical procedure, its potential for monitoring is limited since it is not feasible to continuous analyze mutated genes. Additionally, sampling tumor tissue is subject to selection bias resulting from tumor heterogeneity (6).

Analysis of circulating tumor DNA (ctDNA) is a non-invasive liquid biopsy method to longitudinally monitor the NSCLC progression and response to therapy (7, 8). Although plasma ctDNA is commonly used for detecting somatic mutations derived from malignant tumors, limitations exist on the uncertainty of collection, and processing methods and its detection performance in diversity of mutation phenotypes (9).

Other biofluids such as saliva could provide alternative sources for ctDNA assessment. Saliva is the epitome of a non-invasive, readily-available, and easily-collectable biofluid, and is more suitable for serial detections of ctDNA than blood. Existing ctDNA detection platforms, such as droplet digital PCR (ddPCR) and next-generation sequencing (NGS), are complicated, technique dependent, time consuming, and has demonstrated limited success in saliva ctDNA detection (10). We have developed an electric field–induced release and measurement (EFIRM) liquid-biopsy technology to detect and quantify the ctDNA in 50 μL of saliva of NSCLC patients with near perfect concordance with biopsy genotyping (96–100%) (11–13). Thus, the saliva-based EFIRM could be an effective platform for longitudinal monitoring of gene mutations in a clinical setting to influence changes in therapy.

In the present study, we report on two advanced NSCLC patients with the treatment of osimertinib before and after local ablative therapy (LAT), who underwent longitudinal monitoring of both *EGFR* and *PIK3CA* mutations using the saliva-based EFIRM platform. The major aims of this case report were the following:

1. Determine the ability to monitor the expressions and changes of *EGFR* and *PIK3CA* mutations in advanced NSCLC by saliva-based EFIRM platform.
2. Determine if statistically combining both *EGFR* and *PIK3CA* ctDNA mutations will enhance the ability to longitudinally monitor the disease progression in NSCLC by saliva-based EFIRM.

## Patients and Methods

### Monthly Serial Collections of Saliva and Plasma as Well as Regular Evaluation of Tumor Volume by CT

From May 2016 through September 2017, two patients histologically diagnosed with unresectable advanced NSCLC were enrolled into National Institutes of Health Clinical Center (NIHCC) clinical trial (ClinicalTrials.gov: 16-C-0092; local ablative therapy for the treatment of oligoprogressive, *EGFR*-mutated, non-small cell lung cancer after treatment with osimertinib). For each patient, serial blood and saliva samples were collected at day 0 (baseline), day 7 of cycle 1, and the 1st day of each cycle thereafter. Initially, a cycle of osimertinib treatment was set for 21 days for patients but was later amended to 28 days. Patients took osimertinib until they could no longer tolerate it or their disease worsens. The method of plasma and saliva collection protocol are described in Supplementary Materials. The study was approved and performed under the supervision of the NIHCC and the University of California, Los Angeles (UCLA) Institutional Review Board. Written informed consent was obtained from all participants. Each patient met the inclusion criteria of the clinical trial which are provided in Supplementary Materials.

All target lesions were identified at each tumor assessment using CT every 6 weeks. In order to estimate of the tumor burden in the body, all other metastatic soft-tissue lesions ≥ 10 mm in long-axis diameter were selected for analysis. For lymph nodes, lesions ≥ 15 mm in short axis were considered. All such lesions were manually segmented and the volume was measured using Carestream PACS (Vue PACS version 12.1, Carestream Health, Rochester, New York). Lesion volumes were assessed even if the volume got smaller in the follow-up scans. Tissue samples derived from primary and metastasized tumors were evaluated according to the routine procedures by the National Cancer Institute (NCI) Laboratory of Pathology.

### Saliva-Based EFIRM Detection for *EGFR* and *PIK3CA* Mutations

Serial saliva samples were used to monitor p.L858R and *exon19 del EGFR* mutations and p.E545K *PIK3CA* by the EFIRM platform (EZLife Bio, Woodland Hills, CA), which is an open platform signal amplification technology based on a microtiter plate of 96 gold electrodes obtained from EZLife Bio (Woodland Hills, CA), China). The basic EFIRM assay has been described previously (11, 12). Of note is that each measurement is performed in duplicate as part of the standard operating procedure. Paired probes (capture and detector probes; Integrated DNA Technologies, San Diego, CA) specific for three included mutations in NSCLC were designed are listed in Table 1.

**Table 1:** Probe sequences for p.L858R *EGFR, exon 19 del EGFR*, and p.E545K *PIK3CA*.

**Mutation**		**Probe sequence**
EGFR L858R	Capture probe	5′-TGG CCC GCC C-3′
	Detector probe	5′-AAA ATC TGT GAT CTT GAC ATG CTG CGG TGT TTT GTG CAG-3′
EGFR exon 19 deletion	Capture probe	5′-TGT TGC TTC CTT G-3′
	Detector probe	5′-ATA GCG ACG GGA ATT TTA ACT TTC TCA CCT-3′
PIK3CA E545K	Capture probe	5′-TCC TGC TTA GTG AT-3′
	Detector probe	5′-TTC AGA GAG AGG ATC TCG TGT AGA AAT TGC TT-3′

*The detector probes were biotinylated on the 3′ end*.

**Figure 1:**
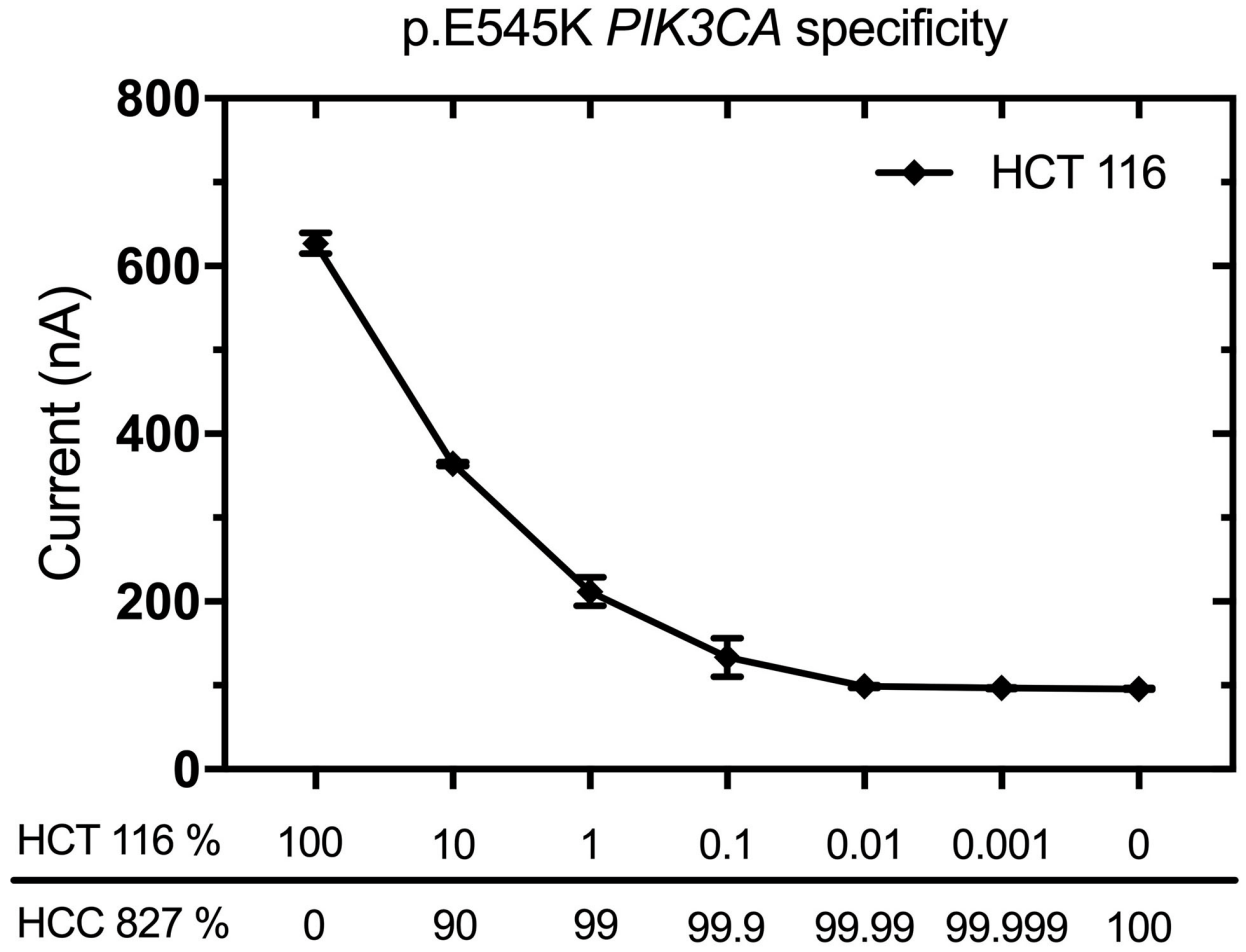
Validation of specificity and sensitivity for p.E545K *PIK3CA* by EFIRM detection using lung cancer cell lines. Wild-type *PIK3CA* (from HCC827 cells) and p.E545K *PIK3CA* (from HTC 116 cells) were assayed by decreasing the ratio of targeted oncogene sequence to other sequences. As little as 0.1% p.E545K mutant DNA was detected when a wide-type sample was used. We used 10 μL of 2 ng/μL DNA for these experiments.

The specificity and sensitivity of EFIRM detection for p.L858R *EGFR* and *exon19 del EGFR* variants have been tested in previous studies (11, 12). In this case report, we also validated the specificity and sensitivity of p.E545K *PIK3CA* mutation by decreasing the ratio of p.E545K *PIK3CA* genomic DNA to wild-type *PIK3CA* genomic DNA, which were from cell line HTC 116 (AccuRef, Milpitas, CA) and HCC827 (AccuRef, Milpitas, CA), respectively. As little as 0.1% p.E545K mutant DNA was detected when a wide-type sample was used (Figure 1). We used 10 μL of 2 ng/μL DNA for these experiments. These data demonstrated that EFIRM was able to detect p.E545K *PIK3CA* mutation with high sensitivity and specificity.

Plasma-based ddPCR assay for p. L858R and *exon19 del EGFR* variants and plasma-based NGS for p.E545K *PIK3CA* mutation were performed as described in Supplementary Materials. In the present study, the data of plasma-based ddPCR and NGS as well as the volumetric tumor analysis of tumors as considered golden-standard for the data of EFIRM assay.

For the analysis of data and curves in tumor volume, ddPCR, NGS, and EFIRM assays, an increase or decrease in ctDNA load is noted only if the values of two subsequent time-points are both higher or lower than the baseline value, and at least a 20% rise or 30% decline to the baseline. The MATLAB 2018R software (MathWorks, Inc., Massachusetts, USA) was used to compare the similarity between different curves by calculating an *R*-value to show the demonstrate similarity between ctDNA load curves. The closer *R*-value is to 1, the stronger the similarity is between two curves. *P* < 0.05 was considered to indicate a statistically significant difference between values.

## Results

### Case Report 1

A patient presented with extra-cranial advanced NSCLC with metastatic liver lesions who previously received palliative chemotherapy with first generation TKIs. They were confirmed to harbor *exon19 del EGFR*, p.E545K *PIK3CA*, and p.T790M *PIK3CA* sensitizing somatic cis-mutations during enrollment. The patient commenced with 80 mg osimertinib per day.

**Figure 2:**
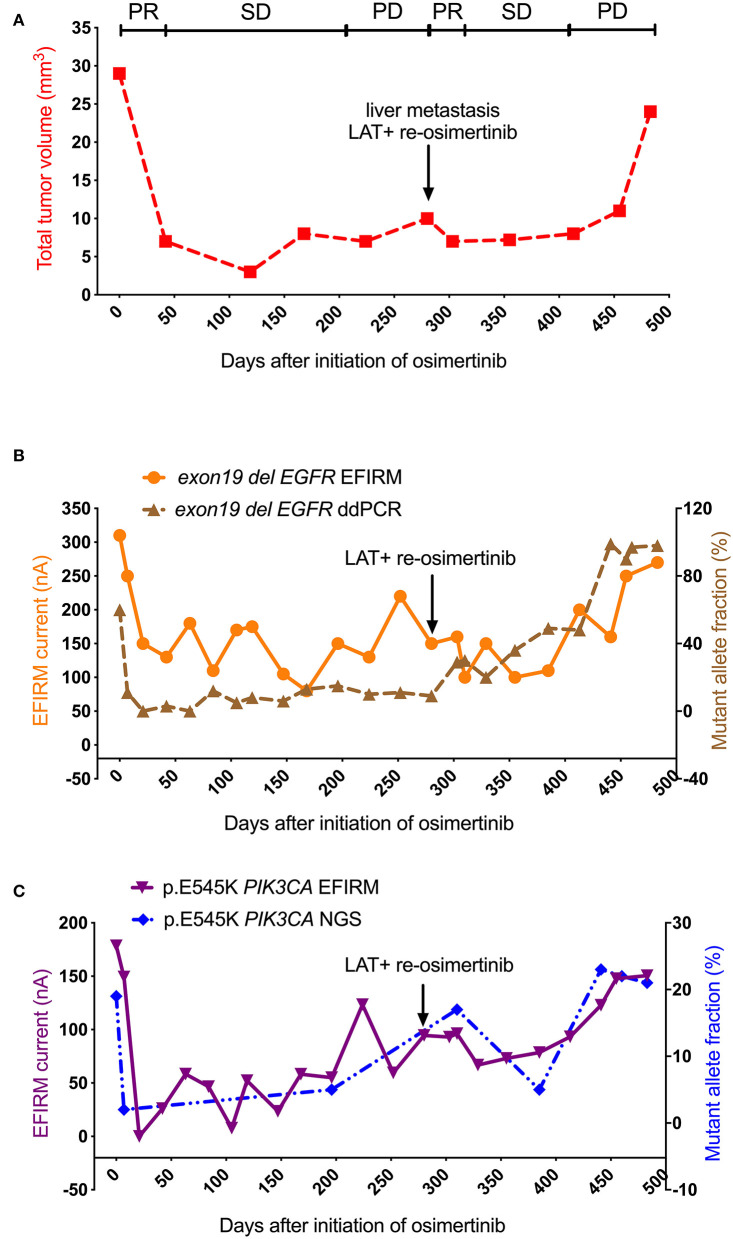
Saliva-based EFIRM for detection of *exon19 del EGFR* and p.E545K *PIK3CA* in Case 1. **(A)** The volume of metastatic tumor showed a sharp decline after the initiation with 80 mg osimertinib. At the time point of day 280 (black arrow), LAT and followed osimertinib re-initiation were performed. The tumor volume increased to a high level eventually. **(B)** The curve of *exon19 del EGFR* EFIRM has a strong similarity to that of *exon19 del EGFR* ddPCR (*R* = 0.53, *P* = 0.01). **(C)** The curve of p.E545K *PIK3CA* EFIRM has a strong similarity to that of p.E545K *PIK3CA* NGS (*R* = 0.72, *P* = 0.00), respectively.

As shown in Figure 2A, after the initiation with osimertinib treatment, there was a sharply partial response (PR) period (from day 0 to 42) for the volume of metastatic tumor (from 30 to 7 mm^3^). The tumor volume entered stable disease (SD) period from day 42 to 224 (from 7 to 7 mm^3^). From day 224 to 280, the volume of metastatic tumor was found to increase more than 20% from 7 to 10 mm^3^, which indicating that the progression of metastatic tumor entered the progressive disease (PD) period. At day 280, the PD was confirmed by the tissue re-biopsy of liver. Local ablative therapy of the metastatic tumor was performed followed by the re-initiation of osimertinib. This combined therapy resulted directly in the second PR, and one-third reduction (from 10 to 7 mm^3^) of the metastatic tumor volume in the following 23 days, and kept the tumor volume in a SD period (from day 303 to 413) for 110 days. However, the tumor volume increased significantly again at its second PD period (from day 413 to 483), and reached its second highest volume at three times the lowest volume after LAT and subsequently, the patient passed away.

As shown in Figure 2B, in the sharply PR period of tumor volume, *exon19 del EGFR* ctDNA detected by both plasma-based ddPCR and saliva-based EFIRM was also markedly decreased (ddPCR from 60 to 3%; EFIRM from 310 to 150 nA). After that, in the SD period, *exon19 del* ctDNA by ddPCR analysis fluctuated up and down at the low levels from day 21 to 280. However, *exon19 del* ctDNA load in EFIRM went on to decrease to its lowest point from day 21 to 168 (from 150 to 80 nA). Then, in the PD period, *exon19 del* analysis by EFIRM appeared to suddenly rise to three-fold the ctDNA load in 52 days. After LAT at day 280, unlike like the stable period of tumor volume, there was a continuous increase of ddPCR mutant allele fraction (from 9 to 84%) of *exon19 del* in next 161 days. On the contrary, after LAT treatment *exon19 del* ctDNA analysis by EFIRM demonstrated a gradual decrease to the two-thirds of signal at day 280 in the second PR, before starting to rise again. Eventually both signals of ddPCR and that of EFIRM reached their rapidly increasing periods, mirroring the behavior of the tumor volume.

As shown in Figure 2C, p.E545K *PIK3CA* mutant tested by both plasma-based NGS and saliva-based EFIRM also decreased sharply in the PR period of tumor volume after the initiation of osimertinib (NGS from 19 to 2%; EFIRM from 179 to 0 nA). Then, both started to rise slowly during the SD period of tumor metastatic. After LAT, p.E545K tested by NGS was found a decline to its second lowest point at day 385, followed a sharp increase to its highest point in the second PD period of tumor volume. Moreover, p.E545K ctDNA load EFIRM analysis underwent a slight decline from day 280 to 329, then started to increase significantly until to the end in the second PD period.

After comparing the similarities between different curves, the curve of *exon19 del EGFR* EFIRM demonstrated a strong correlation to the volumetric tumor curves (*R* = 0.78, *P* = 0.00) and *exon19 del EGFR* ddPCR (*R* = 0.53, *P* = 0.01). The curve of p.E545K *PIK3CA* EFIRM has a strong similarity to curves of tumor volume (*R* = 0.70, *P* = 0.00), *exon19 del EGFR* ddPCR (*R* = 0.72, *P* = 0.00) and p.E545K *PIK3CA* NGS (*R* = 0.72, *P* = 0.00), respectively. Additionally, the *exon19 del EGFR* EFIRM curve is similar to that of p.E545K *PIK3CA* EFIRM curve (*R* = 0.65, *P* = 0.00).

### Case Report 2

The second patient presented with histologically confirmed advanced NSCLC (extra-cranial metastases) with metastatic nodes. This patient had no prior *EGFR* TKI therapy. The activating cis-isomerism mutations of p.L858R *EGFR* and p.E545K *PIK3CA* were confirmed by the initial molecular genetic examination of tumor tissue. When enrolled in the NIHCC study, the patient commenced on the treatment with 80 mg osimertinib per day.

**Figure 3:**
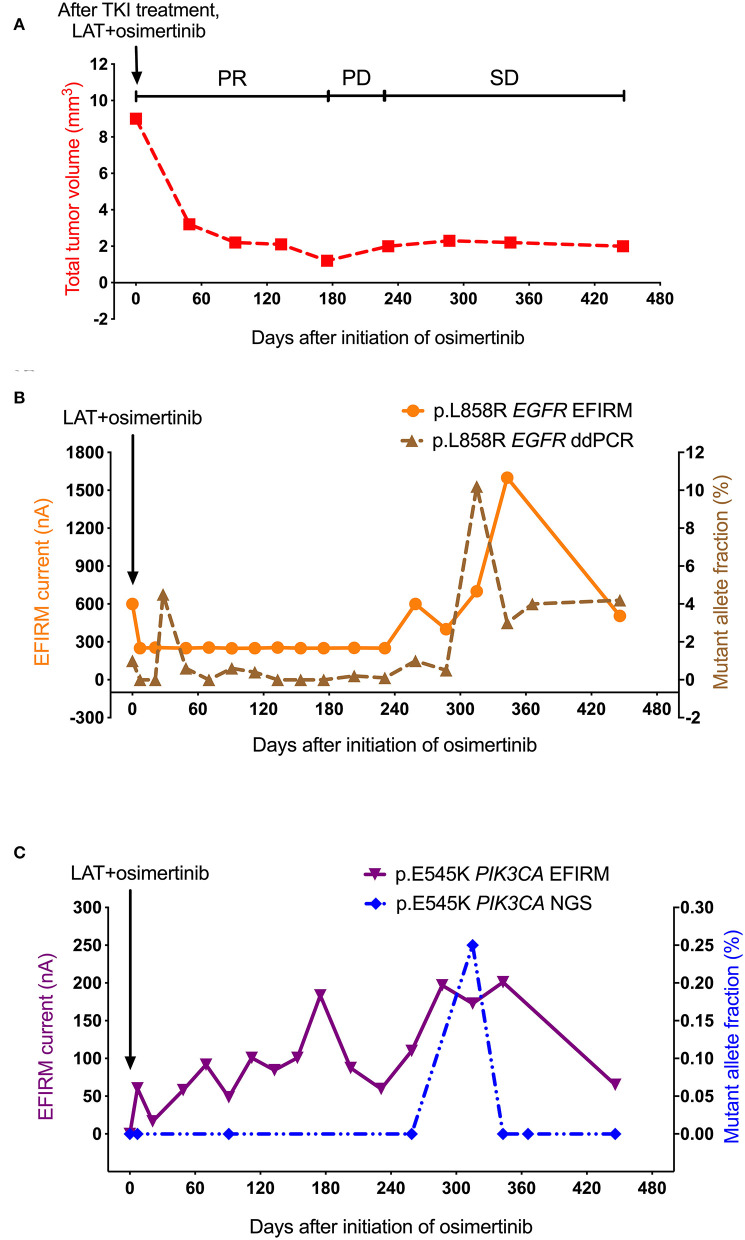
Saliva-based EFIRM for detection of p.L858R *EGFR* and p.E545K *PIK3CA* in Case 2. **(A)** The volume of metastatic tumor showed an obvious decrease after LAT and initiation with 80 mg osimertinib. **(B)** The change trends of p.L858R *EGFR* EFIRM data have significant similarities with plasma-based ddPCR for p.L858R *EGFR* (*R* = 0.467, *P* = 0.04). **(C)** The p.E545K *PIK3CA* tested by plasma-based NGS remained undetectable till to day 315, which showed the only and unapparent peak (from 0 to 0.2%). On the contrary, the EFIRM signal of p.E545K *PIK3CA* mutation increased gradually from the beginning.

As shown in Figure 3A, the PR with regression of metastatic tumor size was found after the treatment of LAT and osimertinib. Tumor volume reached its lowest point at day 175. After, tumor size began to increase slowly in its PD period, but keep at low sizes. Then, tumor volume entered the SD period. However, this patient still passed away eventually.

As shown in Figure 3B, accompanying changes in the PR period of tumor volume, p.L858R *EGFR* mutation detected by both plasma-based ddPCR and saliva-based EFIRM demonstrated a brief decline, then remained at an undetectable level until day 287. However, only at the late stage of PD period, p.L858R *EGFR* mutation detected by both ddPCR and EFIRM began to increase, which were both at a later timepoint than the detected volume increase of tumor by CT in the PD period. Contrasting the tumor volume in the SD period, p.L858R *EGFR* signals of both platforms started to sharply rise in the SD stage of tumor (ddPCR from 0.5 to 10.2% in 28 days; EFIRM from 400 to 1,600 nA in 56 days), then dropped sharply after.

As shown in Figure 3C, the allele fraction of p.E545K *PIK3CA* mutation tested by NGS remained undetectable till to day 315 in the SD period of tumor, at which showed the only peak of signal (from 0 to 0.2%). On the contrary, the EFIRM signal of p.E545K *PIK3CA* mutation did not sharply decrease in the PR period of tumor volume, instead increasing gradually from the baseline. Eventually, p.E545K *PIK3CA* tested by EFIRM reached its highest peak at the middle stage of SD period of tumor, which was followed by a 2-month decline.

Only the EFIRM data of p.E545K *PIK3CA* demonstrated a significantly reversed correlation to the curve of tumor volume data (*R* = −0.65, *P* = 0.01). Meanwhile, p.L858R *EGFR* EFIRM data had significant similarities with p.E545K *PIK3CA* EFIRM detection (*P* = 0.00) and p.L858R *EGFR* ddPCR (*P* = 0.04), although the correlation was not very strong (*R* = 0.50 and 0.47, respectively).

## Discussion

Circulating tumor DNA (ctDNA) has emerged as a promising non-invasive tool to detect various genomic alterations associated with NSCLC, which could be used to monitor TKI treatment effectiveness, detect “druggable” mutations and improve prognosis (14). Even for NSCLC patients with metastases, *EGFR* ctDNA can be an independent prognostic factor that can be monitored in the biofluid (15).

EFIRM is a new development for ctDNA detection in plasma or saliva. It utilizes an electric field to enhance hybridization efficiency and detect perfect duplexes. In comparison with predominantly PCR-based platforms, EFIRM is performed directly on unprocessed biofluid samples without the need for DNA isolation, adaptor ligation, bead purification, and sample manipulation. Moreover, EFIRM platform can also be transformed into high-throughput oncogenic mutation analysis lab assays for rapidly identifying oncogenic mutations. The advantages of EFIRM can enable clinicians to adjust their therapeutic strategies in a timely and easy fashion, consequently improving the clinical therapy response.

Rapid advances have been made in the field of salivary diagnostics because saliva contains already established omics molecular biomarkers reported in blood and urine. These biomarkers can be used in early detection and monitoring of various types of cancer (16). By using capability of continuous analysis of the saliva-based EFIRM platform, this study presents two different advanced NSCLC patients who received longitudinal monitoring of disease progression. The drift curve of EFIRM signals of *EGFR* mutations and that of the tumor volume evaluated by CT were concordant in these two. Although in the second case EFIRM analysis of *PIK3CA* mutation differed from the CT volumetric evaluation, it could be a classic example of the characterization of clonal redistribution: newly detected *PIK3CA* mutation occurred during the anti-*EGFR* TKI treatment, which is resultant from predominant suppression of *PIK3CA* wild-type clones and indirect selection of *PIK3CA* mutated clones (17). Moreover, the ctDNA longitudinal trends of saliva-based EFIRM were also highly correlated with that of plasma-based ddPCR and NGS, which were collected simultaneously during treatment. It is notable that in Case 2, the majority of ctDNA progression was not found by plasma NGS except for only one time point. However, EFIRM detected clear increase and decrease of *PIK3CA ctDNA* load in saliva, which suggests the complementary roles of different ctDNA methodologies in predicting treatment response and resistance to EGFR-TKI therapy. Thus, these results suggest saliva-based EFIRM can complement imaging analyses and plasma-based ddPCR or NGS for accurate and comprehensive reflection of disease progression and response for TKIs treatment in patients with different TKI history. However, the origins of ctDNA in saliva remains unclear. Proteomic studies of saliva revealed that 20-30% of the salivary proteome mirrors the plasma proteome, indicating that a substantial portion of salivary constituents are derived from the blood (18). The significant overlap between saliva and blood due to their physiological interactions supplied more evidences that saliva-based EFIRM could be an alternative approach to detect and monitor the progression of disease compared to plasma-based platforms.

In the present study, *exon19 del* and p.L858R *EGFR* which are the most commonly activated mutations in NSCLC, and p.E545K *PIK3CA* which is a reliable predictor of resistance to *EGFR* TKI as the combined biomarkers were used to continuously monitor the disease progression of two NSCLC cases (19–21). Saliva-based EFIRM platform found that the changes of p.E545K *PIK3CA* had close similarities to that of *exon19 del EGFR* in the case 1, and p.L858R *EGFR* in the second NSCLC case. Therefore, it could be more meaningful for clinicians to longitudinally monitor both *EFGR* and *PIK3CA* mutations at the same time-points in the progressive development of advanced NSCLCs than single *EGFR* mutation.

As well, LAT and osimertinib re-challenge had some practical considerations based on the TKI therapy history of both mutational and tumor loads of each advanced NSCLC patient. On one hand for the NSCLC patient with naive *EGFR* TKI therapy before (Case 2), the first initiation of osimertinib could suppress the expressions of both p.L858R *EGFR* and p.E545K *PIK3CA* mutations simultaneously during the whole disease course. On the other hand, for the patient with AR to first generation TKIs (Case 1), the initiation of osimertinib can decline the *exon19 del EGFR* level at the early course, however there was still found a metastatic liver lesion. Following LAT and retreatment of osimertinib cannot stop the emergence of severe PD at the late course. These results could be supported by the explanation that osimertinib can inhibit *PI3K/Akt* pathway in NSCLC cancer cells *in vitro* and *in vivo*, and the re-activation of *PI3K/Akt* bypass signaling pathways might contribute to the osimertinib AR (22). However, it does not appear to match the observation that inhibition of *EGFR* signaling pathway may activate other bypass signaling pathway because of the mutual substitution between signal transduction pathways (23). The direct initiation of third-generation TKIs for advanced NSCLCs with no prior TKI therapy in the present study could be the reason for the inconsistency between their conclusions and these cases. Moreover, the difference should be validated in our further studies with large samples.

For further improvement of survival benefits in *EGFR* TKI-AR NSCLC patients, many groups have explored the combination of *EGFR* TKI and other targeted agents, including dual *EGFR* blockade and bypass signaling pathway blockade. The combination of two common TKIs, erlotinib and cetuximab, induced tumor regression by reducing the phosphorylation of *EGFR* and subsequent silence of downstream signaling pathway in xenograft *EGFR* TKI-resistant NSCLC mice models (24). However, the activation of bypass signaling pathway warranted proliferation, progression and metastasis to NSCLC prior to *EGFR* TKI because of the crosstalk and mutual substitution between variable signaling pathways. In a phase II clinical trial of combining tivantinib (targeting c-Met) with erlotinib in *EGFR* TKI resistant NSCLC patients, the c-Met high subgroup achieved more survival benefit compared with c-Met low subgroup in terms of median PFS (4.1 vs. 1.4 months) and median OS (20.7 vs. 13.9 months) (25). Other phase Ib/II clinical trial of capmatinib (targeting MET) plus gefitinib in *EGFR* TKI-resistant NSCLC patients, the ORR was 27% and increased activity was corresponded to higher MET amplification (26). Thus, the opposing trend exhibited by *EFGR* and *PIK3CA ctDNA* in the study, may be related to the fact that advanced NSCLCs with both *EFGR* and *PIK3CA* mutations can related to interactions by respective inhibitors at the same time during the disease progression. Some reports found that the combination of other rare mutation inhibitors and taselisib targeting *PI3K* pathways could result in a synergistic effect on NSCLC cell growth inhibition and enhancing the response to TKIs both *in vitro* and *in vivo* (27, 28). Moreover, two multicentered, open-label, non-randomized phase II studies about the combination of *EGFR* TKIs and *PI3K* inhibitor (BKM120) in patients with advanced NSCLC (ClinicalTrials.gov: NCT01487265, NCT01570296) have been under evaluation. However, there is no published report about the combination of *EGFR* TKIs and *PI3K* inhibitors in the targeted therapy of advanced NSCLC until now.

## Conclusion

This case report demonstrates that saliva-based EFIRM is capable of longitudinal monitoring the NSCLC progression and response to adjuvant therapies by the combination of *EGFR* and *PIK3CA* mutations.

## Ethics Statement

The studies involving human participants were reviewed and approved by the NIHCC and University of California, Los Angeles (UCLA) Institutional Review Board. The patients/participants provided their written informed consent to participate in this study.

## Author Contributions

NL, DW, FW, UG, and CK: conception and design of the study. NL, FW, LY, JC, and FL: analysis and interpretation of data. NL, DW, FW, and DC: drafting and revising the article. DW and FW: final approval of the version.

## Conflict of Interest

FW's spouse is the co-founder and CEO of EzLife Bio. Her spouse receives income and holds equity in the company. FW is the co-inventor of intellectual property by the UC regents and being used in this research. The remaining authors declare that the research was conducted in the absence of any commercial or financial relationships that could be construed as a potential conflict of interest.
