# Supplementary material for: Longitudinal Monitoring of EGFR and PIK3CA Mutations by Saliva-Based EFIRM in Advanced NSCLC Patients With Local Ablative Therapy and Osimertinib Treatment: Two Case Reports
Source: Front Oncol. 2020 Jul 24;10:1240. doi: 10.3389/fonc.2020.01240 (PMC7393232; doi:10.3389/fonc.2020.01240)
Supplement: Supplementary file 1 [file Data_Sheet_1.docx]

**Supplementary Materials**

Inclusion criteria of the clinical trial:

1. Harboring *exon19 del* or p.L858R *EGFR* mutations as well as p.E545K *PIK3CA* mutation, because the *exon19 del* and the L858R point mutation represent 90% of EGFR sensitizing mutations.
2. Patients harbored p.T790M *EGFR* mutation.
3. Presence of measurable disease per Response Evaluation Criteria in Solid Tumors (RECIST) version 1.1
4. Eastern Cooperative Oncology Group (ECOG) performance status 0-2.
5. Adequate end organ function.
6. Absolute neutrophil count > 1000 /cu.mm; platelet count > 100,000 /cu.mm.
7. Creatinine clearance > 50 mL/min.
8. AST, ALT, Total bilirubin < 1.5 times upper limit of normal.
9. Without leptomeningeal disease, second malignancy and uncontrolled systemic diseases (hypertension, cardiac disease, diabetes) before enrollment.

Methods and Materials of plasma and saliva collections:

1. Blood was collected in EDTA-containing tubes and stored on ice until processing. Plasma was separated by centrifugation at 4^o^C for 10 minutes at 2000 x *g* and the plasma cleared by centrifugation at 4^o^C for 10 minutes at 14,000 x *g*.
2. Saliva was collected in Pure•SALTM (Oasis Diagnostics, Vancouver, WA) and stored at -80^o^C without further processing.
3. Healthy saliva samples were collected from healthy student donators in UCLA as the negative controls using Pure•SALTM saliva collector (Oasis Diagnostics, Vancouver, WA) at Dr. Wong lab. Saliva collection was at least 30 minutes before or after any food consumption. The sponge component of collector was placed in the vestibule mouth and passively absorb saliva until saturated and processed through the syringe filter into an empty 2mL Eppendorf tube. Saliva was immediately stored at 80^o^C without further processing.

Methods and Materials of ddPCR and NGS

1. ddPCR

According to the manufacturer’s instructions, circulating tumor DNA (ctDNA) was isolated from 2-4 mL plasma samples using the MagMax cell-free DNA isolation kit with the KingFisher Prime Duo instrument (ThermoFisher). ctDNA detection was performed on a BIO-RAD QX200 ddPCR system using the custom PrimePCR ddPCR mutation detection assay (BIO-RAD, Hercules, CA) for *EGFR* mutations. Each PCR reaction contained 10 µL of 2 x ddPCR supermix for probes (no dUTP), 1 µL 20 x mutant primers/probe mix (FAM) and wild type primers/probe mix (HEX) mix, 1 µL nuclease-free water, and 8 µL of cfDNA. The assay was performed in duplicate. The presence of mutant DNA copies and the fractional abundance of the mutant allele were determined with QuantaSoft v.1.7 (BIO-RAD).

1. NGS

The data of the Next Generation Sequencing testing were generated using an amplicon-based sequencing platform (InvisionSeq™-Lung). Briefly, sequencing libraries were created from extracted circulating tumor DNA (ctDNA) using a two-step amplification process and were sequenced on Illumina NextSeq 500 platform. Using a proprietary analytical pipeline, genomic alterations were identified and reported.
